# A Novel Multi-Scale Entropy Approach for EEG-Based Lie Detection with Channel Selection

**DOI:** 10.3390/e27101026

**Published:** 2025-09-29

**Authors:** Jiawen Li, Guanyuan Feng, Chen Ling, Ximing Ren, Shuang Zhang, Xin Liu, Leijun Wang, Mang I. Vai, Jujian Lv, Rongjun Chen

**Affiliations:** 1 School of Computer Science, Guangdong Polytechnic Normal University, Guangzhou 510665, China; lijiawen@gpnu.edu.cn (J.L.); fengguanyuan@gpnu.edu.cn (G.F.); lingchen@gpnu.edu.cn (C.L.); renximing@gpnu.edu.cn (X.R.); wangleijun@gpnu.edu.cn (L.W.); jujianlv@gpnu.edu.cn (J.L.); 2 Key Laboratory of Cognitive Neuroscience and Applied Psychology (Education Department of Guangxi Zhuang Autonomous Region), Guangxi Normal University, Guilin 541004, China; 3 ZUMRI-LYG Joint Laboratory, Zhuhai UM Science and Technology Research Institute, Zhuhai 519031, China; fstmiv@um.edu.mo; 4 School of Artificial Intelligence, Neijiang Normal University, Neijiang 641004, China; 5 School of Life Science and Technology, University of Electronic Science and Technology of China, Chengdu 610056, China; 6 State Key Laboratory of Digital Medical Engineering, Southeast University, Nanjing 211189, China; 7 School of Mathematics and Computer Science, Northwest Minzu University, Lanzhou 730030, China; xinliu2024@xbmu.edu.cn; 8 Department of Electrical and Computer Engineering, University of Macau, Macau 999078, China; 9 Guangdong Provincial Key Laboratory of Intellectual Property and Big Data, Guangdong Polytechnic Normal University, Guangzhou 510665, China

**Keywords:** electroencephalography (EEG), multi-scale entropy, lie detection, machine learning, channel selection

## Abstract

Entropy-based analyses have emerged as a powerful tool for quantifying the complexity, regularity, and information content of complex biological signals, such as electroencephalography (EEG). In this regard, EEG-based lie detection offers the advantage of directly providing more objective and less susceptible-to-manipulation results compared to traditional polygraph methods. To this end, this study proposes a novel multi-scale entropy approach by fusing fuzzy entropy (FE), time-shifted multi-scale fuzzy entropy (TSMFE), and hierarchical multi-band fuzzy entropy (HMFE), which enables the multidimensional characterization of EEG signals. Subsequently, using machine learning classifiers, the fused feature vector is applied to lie detection, with a focus on channel selection to investigate distinguished neural signatures across brain regions. Experiments utilize a publicly benchmarked LieWaves dataset, and two parts are performed. One is a subject-dependent experiment to identify representative channels for lie detection. Another is a cross-subject experiment to assess the generalizability of the proposed approach. In the subject-dependent experiment, linear discriminant analysis (LDA) achieves impressive accuracies of 82.74% under leave-one-out cross-validation (LOOCV) and 82.00% under 10-fold cross-validation. The cross-subject experiment yields an accuracy of 64.07% using a radial basis function (RBF) kernel support vector machine (SVM) under leave-one-subject-out cross-validation (LOSOCV). Furthermore, regarding the channel selection results, PZ (parietal midline) and T7 (left temporal) are considered the representative channels for lie detection, as they exhibit the most prominent occurrences among subjects. These findings demonstrate that the PZ and T7 play vital roles in the cognitive processes associated with lying, offering a solution for designing portable EEG-based lie detection devices with fewer channels, which also provides insights into neural dynamics by analyzing variations in multi-scale entropy.

## 1. Introduction

Lie detection technology holds significant applications across judicial, security, and psychological domains [1]. Conventional methods, which typically include the strategic usage of evidence, familiarity with the context, diagnostic questioning, instrument-aided physiological and behavioral monitoring, contextual examination, repeated interrogation, expert analysis, intuitive processing, and motivational inference, rely on personal experience [2]. Currently, while polygraphs can infer an individual’s deceptive state by measuring physiological changes such as respiration, heart rate, and skin galvanic response, their reliability should not be overestimated, as the accuracy of polygraph results closely depends on the abilities and knowledge of the individual being tested. Additionally, specific individuals under the influence of anesthetics, alcohol, and drugs remain unsuitable for examinations [3]. Thus, lie detection using machine learning methods has garnered interest due to its enhanced objectivity and efficiency.

Previously, Opancina et al. [4] mentioned that combining interview patterns with neuroimaging techniques, such as functional magnetic resonance imaging (fMRI), can be used for lie detection. Nonetheless, fMRI relies on detecting brain metabolic activities, rendering it time-consuming in practice and unfavorable for designing portable systems [5,6]. In contrast, electroencephalography (EEG), a non-invasive way for investigating brain activities, only requires electrodes placed on the scalp to collect biological signals, providing better operability and safety. More importantly, EEG signals exhibit high temporal resolution and relatively low cost, facilitating the real-time recording of neural variations during cognitive, emotional, and decision-making processes in the brain [7]. Meanwhile, related works [8,9] found that when an individual lies, the brain engages in inhibiting truthful information and constructing false information, which can be viewed as neurophysiological biomarkers in EEG signals for lie detection.

Generally, to obtain valuable characteristics pertinent to lie detection from EEG signals, feature extraction is an essential step. Traditional approaches primarily focus on time-domain and frequency-domain analysis. As for the time domain, extensively used features include amplitude, latency, and waveform morphology, such as components of event-related potentials (ERPs) [10]. Specifically, P300 amplitude and latency, which are associated with attention allocation and information updating, have been employed in this field [11]. However, time-domain features are often susceptible to noise and struggle to denote the dynamic changes of the EEG signals [12]. Frequency-domain techniques enable the extraction of power spectra and phase features from different frequency bands that reflect various cognitive states [13,14,15]. Furthermore, entropy-based analyses have emerged as a powerful solution for quantifying the complexity, regularity, and information content of EEG signals [16]. Nevertheless, traditional single-scale entropy measures, such as approximate entropy, permutation entropy, and sample entropy, are insufficient for identifying biomarkers of EEG signals across different time or frequency scales. This limitation indicates that crucial dynamic information related to lie detection is missed if the analysis is restricted to a single scale. Hence, the multi-scale entropy approach is more appropriate for investigating complex brain activities under diverse conditions. Regarding the frequency-domain multi-scale entropy, Yu et al. [17] used Butterworth filters to decompose EEG signals into four frequency bands for emotion recognition. Then, they extracted differential entropy, nonlinear entropy, and maximum entropy from each frequency band to achieve good performance through a multi-band approach. Niu et al. [18] investigated the characteristics of multi-band by combining Shannon-based multivariate permutation entropy and conditional-based multivariate sample entropy, offering a perspective for quantifying complex EEG dynamics. Chen et al. [19] extract a series of brain maps based on spatial features to form frequency-domain entropy for analyzing EEG signals.

Concerning multi-scale entropy, they helpfully address the issue of information loss in traditional coarse-graining methods. For example, Shang et al. [20] claimed the drawback of coarse-graining methods, where practical information diminishes with increasing scale, leading to reduced entropy stability. Therefore, the concept of time shift was applied by proposing the time-shifted multi-scale increment entropy, which utilizes the property of Higuchi fractal dimension to generate a new set of time series with fractal curve characteristics on the original time scale. It not only thoroughly investigates the irregularity of time series data but also preserves vital structural information. Fan et al. [21] found that utilizing the numerical features of dispersion patterns as a new metric makes it proper for quantifying fuzzy dispersion entropy singularity. Their experiments revealed that combining amplitude-based time-shifted coarse-graining methods with fuzzy dispersion entropy provides valuable monitoring indicators for multi-scale calculations. Zheng et al. [22] reported that traditional slope entropy only analyzes time series data at a single scale, neglecting vital information at other scales. Therefore, they developed an improved time-shifted multi-scale slope entropy to enhance classification performance. As discussed, the aforementioned multi-scale entropy approaches facilitate the analysis of signal complexity and dynamics, laying the groundwork for EEG-based lie detection.

Recently, deep learning has brought advantages to EEG-based lie detection, with its automatic high-level feature learning capability [23,24]. Convolutional neural networks (CNNs) and long short-term memory (LSTM) networks are two widely used models in this field. For instance, Saryazdi et al. [25] converted 1D EEG signals into 2D images and then utilized a deep CNN (DCNN) model to achieve lie detection with good results. Aslan et al. [26] employed the discrete wavelet transform (DWT) and min-max normalization preprocessing on EEG signals, subsequently designing an LSTMNCP model that combines LSTM and neural circuit policy (NCP), where the NCP replaces the traditional fully connected layers in LSTM to achieve better computational efficiency and reduced neuron consumption. Rahmani et al. [27] combined a deep graph convolutional network (GCN) with type-2 fuzzy sets (TF-2), acquiring over 90% accuracy in lie detection. Additionally, the V-TAM model proposed by AlArfaj and Mahmoud [28] realized lie detection by fusing spatiotemporal features of EEG signals.

Despite progress in deep learning for EEG-based lie detection, most approaches tend to utilize multi-channel input, which increases data complexity and poses challenges for device portability, as multi-channel data sources often result in redundant data, computational burden, and increased hardware requirements. For example, a 64-channel EEG system makes it challenging to deploy outside of a laboratory setting. That means it is necessary to perform channel selection to investigate representative channels, where the fewer the channels, the fewer electrodes need to be placed on the scalp.

A review of previous works reveals several persistent challenges that hinder the practical application of EEG-based lie detection. First, many high-performing methods rely on a large number of EEG channels (often 16 or more), which limits their potential for portable, real-world applications. Second, conventional feature extraction techniques, particularly single-scale entropy and traditional coarse-graining methods, usually suffer from information loss, potentially overlooking subtle neural markers of deception. Ultimately, the current trend towards computationally intensive deep learning models often overlooks the need for lightweight and efficient systems. Hence, this study is specifically designed to address these gaps by proposing a multi-scale entropy feature that is effective even with a single channel, paving the way for a more practical EEG-based lie detection solution.

To this end, since the multi-scale entropy approach shows potential for analyzing complex EEG signals, this study proposes a novel method that fuses fuzzy entropy (FE), time-shifted multi-scale fuzzy entropy (TSMFE), and hierarchical multi-band fuzzy entropy (HMFE), enabling the multidimensional characterization of EEG signals. Then, with the help of machine learning classifiers, the multi-scale entropy feature vector from the channels selected can achieve a satisfactory accuracy in lie detection. Particularly, the main contributions of this study are presented as follows:

1. A novel multi-scale entropy approach is proposed for EEG-based lie detection, which innovatively fuses fuzzy entropy (FE) with time-shifted multi-scale fuzzy entropy (TSMFE) and hierarchical multi-band fuzzy entropy (HMFE) to achieve a multidimensional characterization of EEG signals, overcoming limitations of the existing single-scale methods.
2. A systematic channel selection analysis is conducted to identify the most representative EEG channels for lie detection, which provides an empirical basis for designing portable, few-channel lie detection devices, helpfully reducing hardware complexity.
3. A comprehensive performance evaluation is performed using various machine learning classifiers (SVM, kNN, NB, and LDA), including both subject-dependent and cross-subject validation schemes to assess the proposed method’s effectiveness and generalization capability rigorously.

The remainder is organized as follows: Section 2 describes the proposed multi-scale entropy approach and the classifiers applied, along with the dataset used for evaluation. Section 3 shows the results from the subject-dependent experiment and the cross-subject experiment, as well as the channel selection. Section 4 conducts a comparative study with existing works and discusses the findings. Finally, Section 5 concludes this study and outlines future work.

## 2. Proposed Method

**Figure 1:**
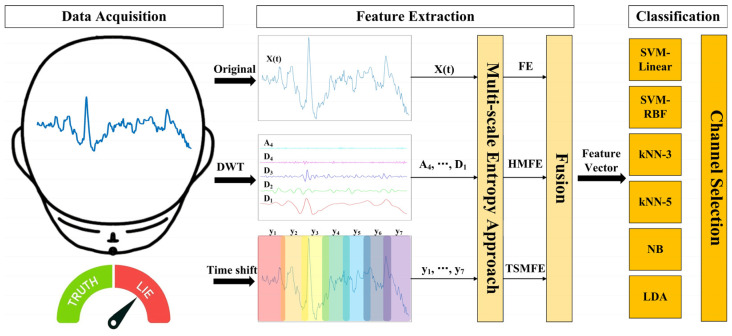
Overall flowchart of the proposed EEG-based lie detection method, where different colors in the time shift represent various time-shifted sequences.

For better understanding, Figure 1 illustrates the overall flowchart of the proposed method, which primarily involves data acquisition, feature extraction, and classification, along with channel selection. More details are described as follows:

### 2.1. Feature Extraction

Single-scale entropy is insufficient to describe the intricate characteristics of EEG signals across diverse temporal or frequency scales. Therefore, this study focuses on the multi-scale entropy approach, where feature extraction of FE is performed first and then develops into two variants: TSMFE and HMFE, which can quantify multi-dimensional characterization to provide more discriminative entropy-based analyses for EEG-based lie detection.

#### 2.1.1. Fuzzy Entropy (FE)

FE represents similarity within a time series as a fuzzy membership degree [29], which circumvents information loss that might arise from rigid thresholding and provides a stable and continuous measurement of complexity. For extracting FE from a given input time series like EEG signals, three parameters are needed: the embedding dimension *m*, which determines the length of the pattern vectors, the tolerance *r*, which controls the fuzziness of similarity, and the fuzzy factor *n*, which defines the shape of the membership function.

Initially, a series of overlapping *m*-dimensional embedded vectors is constructed from the original EEG signals to capture pattern information within local regions. This vector is represented as (1):

(1) $$X_{i}^{m}=\{x_{i},\;x_{i+1},\;\ldots ,\;x_{i+m-1}\},\;i=1,\;2,\;\ldots ,\;N-m+1$$

where $X_{i}^{m}$ denotes the *i*-th *m*-dimensional pattern vector starting from time point *i*, and a total of *N* – *m* + 1 vectors are constructed.

Next, the distance between any two *m*-dimensional vectors, $X_{i}^{m}$ and $X_{j}^{m}$, needs to be calculated. The Chebyshev distance is used in this context, as it sensitively captures the maximum difference at any corresponding position within the pattern vectors. In EEG signals analysis, even if most time points match well, a few significantly deviating points indicate diverse patterns. Therefore, the Chebyshev distance directly reflects this worst-case dissimilarity, aiding in the identification of abrupt changes in the signals, expressed as (2):

(2) $$d_{\mathrm{ij}}^{m}=\underset{k\;=\;0,\;1,\;\ldots ,\;m-1}{\max}\left|x_{i+k}-x_{j+k}\right|$$

where $d_{\mathrm{ij}}^{m}$ means the maximum difference between two *m*-dimensional pattern vectors.

Subsequently, to convert the distances between pattern vectors into a measure of similarity, a fuzzy membership function (*μ*) is applied, which maps distance values within the range [0, 1], representing the degree of similarity between them. This choice not only provides a smooth and continuous decay of similarity, avoiding abrupt changes and information loss caused by threshold selection in traditional binarization methods, but also results in a rapid exponential decrease in membership values as the distance $d_{\mathrm{ij}}^{m}$ increases, which implies that only very close patterns are considered highly similar. Its mathematical expression is (3):

(3) $$\mu \left(d_{\mathrm{ij}}^{m},\;r,\;n\right)=\exp\left(-\frac{{\left(d_{\mathrm{ij}}^{m}\right)}^{n}}{r}\right)$$

The pattern matching degree ${\;\phi }^{m}\left(r,\;n\right)$ is then calculated, which reveals the average similarity of all possible pairs of *m*-dimensional pattern vectors, denoted as (4):

(4) $$\phi^{m}\left(r,\;n\right)=\frac{1}{N-m+1}\sum_{i=1}^{N-m+1}\left[\frac{1}{N-m}\sum_{j=1,\;j\neq i}^{N-m+1}\mu \left(d_{\mathrm{ij}}^{m},\;r,\;n\right)\right]$$

Specifically, this matching degree can be interpreted as the average probability that two *m*-dimensional patterns randomly drawn from the original signals match each other, representing the probability of their similarity.

The physical significance of FE lies in the observation that if a time series exhibits higher complexity, the similarity of its patterns will decrease more significantly when transitioning from *m* to *m* + 1 dimensions, resulting in a higher value. Conversely, if the series is more regular and predictable, the FE will be lower. Therefore, the final FE value also requires the participation of $\phi^{m+1}\left(r,\;n\right)$, expressed as (5):

(5) $$\mathrm{FE}\left(m,\;r,\;n\right)=\ln\phi^{m}\left(r,\;n\right)-\ln\phi^{m+1}\left(r,\;n\right)$$

#### 2.1.2. Time-Shifted Multi-Scale Fuzzy Entropy (TSMFE)

TSMFE draws upon the concept of time-shifted sequence analysis. By constructing multiple time-shifted subsequences across different time scales, it circumvents the potential information loss that occurs during traditional coarse-graining processes. For an EEG signal $\{x_{i}{\}}_{i=1}^{N}$, extracting TSMFE requires presetting the embedding dimension *m*, tolerance *r*, fuzzy factor *n*, and maximum time interval scale factor $k_{\max}$. Notably, the parameters for all entropy calculations are set based on common practices in biomedical signal processing and the preliminary evaluations. The embedding dimension *m* is set to 2, and the fuzzy factor *n* is set to 2. The tolerance *r* is set to 0.15 times the standard deviation of the corresponding time series, which allows for adaptive thresholding based on signal amplitude. For the multi-scale analysis, the maximum scale factor *k*_max_ is set to 10. These values are chosen since they have been widely established in the literature for providing a robust measure of complexity in EEG signals, while also balancing feature effectiveness with computational efficiency.

First, *k* time-shifted subsequences are constructed, which have different starting points but the same sampling intervals to prevent potential smoothing of signals. Each time-shifted subsequence $y_{j}^{\left(k\right)}$ begins from the *j*-th data point of the original signals and is sampled with a step size of *k*, denoted as (6) and (7):

(6) $$y_{j}^{\left(k\right)}=\{x_{j},{\;x}_{j+k},\;x_{j+2k},\;\ldots \},\;j=1,\;2,\;\ldots ,\;k$$

(7) $$kL_{j}^{\left(k\right)}=\left\lfloor \frac{N-j}{k}\right\rfloor +1$$

where $L_{j}^{\left(k\right)}$ indicates the total number of data points that can be extracted starting from the *j*-th point of the original sequence with a step size of *k*. Usually, the length of each subsequence is at least *m* + 1 for calculation. Then, the FE value for each of the *k* time-shifted subsequences $y_{j}^{\left(k\right)}$ is calculated according to (5), as shown in (8):

(8) $$FE_{j}^{\left(k\right)}=\mathrm{FE}\left(y_{j}^{\left(k\right)},\;m,\;r,\;n\right),\;j=1,\;2,\;\ldots ,\;k$$

where $FE_{j}^{\left(k\right)}$ represents the FE value of the *j*-th time-shifted sequence at scale *k*. If a subsequence’s length is insufficient or if the FE calculation fails, it is 0 to prevent unreasonable influence on subsequent average values.

Next, at each scale *k*, the TSMFE is defined as the average of the FE values of all *k* time-shifted subsequences, which facilitates that the complexity measure at each scale fully considers all possible time-shifted starting points at that scale as (9):

(9) $$\mathrm{TSMFE}\left(k\right)=\frac{1}{k}\sum_{j=1}^{k}FE_{j}^{\left(k\right)}$$

By iterating all scales, a TSMFE feature vector is obtained, which comprehensively describes the variation trend of the EEG signals across multiple time scales, providing multi-scale time-domain characterization (10):

(10) $$\mathrm{TSMFE}=\left[\mathrm{TSMFE}\left(1\right),\;\mathrm{TSMFE}\left(2\right),\;\ldots ,\;\mathrm{TSMFE}\left(k_{\max}\right)\right]$$

#### 2.1.3. Hierarchical Multi-Band Fuzzy Entropy (HMFE)

Different frequency bands of EEG signals are associated with distinct cognitive states and physiological processes that occur in the brain. For example, delta bands (0–4 Hz) are typically associated with deep sleep states, theta bands (4–8 Hz) with memory and emotional processing, alpha bands (8–13 Hz) with relaxed states, and beta bands (13–30 Hz) with active thinking and attention. To obtain a more comprehensive understanding of different frequency bands, HMFE is proposed by decomposing the original signals into different frequency bands through DWT [30] and then calculating the FE feature separately for each one.

Technically, extracting the HMFE feature requires specifying embedding dimension *m*, tolerance *r*, fuzzy factor *n*, the selected wavelet basis *ψ*, and the decomposition level *L*. First, the *x*(*t*) is decomposed into *L* layers of frequency band coefficients using the DWT, which conducts decomposition at different frequency resolutions through a series of orthogonal filters, yielding coefficients across various frequency bands, represented as (11):

(11) $$\mathrm{DW}T_{L}\left(x\right)=\{A_{L},{\;D}_{L},{\;D}_{L-1},\;\ldots ,{\;D}_{1}\}$$

where *A_L_* means the *L*-th level approximation coefficients, corresponding to the lowest frequency components of the EEG signals, *D_i_* denotes the *i*-th level detail coefficients, corresponding to the components in different high-frequency bands, where *i* = 1, 2, …, *L*. Each level of detail coefficients corresponds to a specific frequency band, ordered from high to low.

After obtaining the DWT coefficients, it is necessary to reconstruct the time-domain detail corresponding to each frequency band through inverse DWT (IDWT). During this process, only the coefficients of the current band are retained, while coefficients from others are set to zero, ensuring that each reconstructed signal (${\tilde{x}}_{A_{L}}$ and ${\tilde{x}}_{D_{i}}$) contains information only from its specific range (12) and (13):

(12) $${\tilde{x}}_{A_{L}}=\mathrm{IDWT}\left(A_{L},\;0,\;\ldots ,\;0\right)$$

(13) $${\tilde{x}}_{D_{i}}=\mathrm{IDWT}\left(0,\;\ldots ,\;0,\;D_{i},\;0,\;\ldots ,\;0\right),\;i=1,\;2,\;\ldots ,\;L$$

where ‘0’ indicates that the coefficients for the corresponding frequency bands are set to zero. In this study, the ‘wrcoef’ function provided by MATLAB R2023b is used to reconstruct specific levels of approximation or detail coefficients from the DWT. Furthermore, to quantify the complexity of each frequency band, and considering that the energies of different frequency bands may vary significantly, an adaptive adjustment is applied to $r_{A_{L}}$ and $r_{D_{i}}$ for each reconstructed band, based on the standard deviation of each band $\sigma \left({\tilde{x}}_{A_{L}}\right)$ and $\sigma \left({\tilde{x}}_{D_{i}}\right)$, and the standard deviation of the original signal $\sigma \left(x\right)$. It can avoid issues where the same tolerance leads to insufficient sensitivity for low-energy bands or excessive sensitivity for high-energy bands (14) and (15):

(14) $$r_{A_{L}}=r\;\cdot \frac{\sigma \left({\tilde{x}}_{A_{L}}\right)}{\sigma \left(x\right)}$$

(15) $$r_{D_{i}}=r\;\cdot \frac{\sigma \left({\tilde{x}}_{D_{i}}\right)}{\sigma \left(x\right)}$$

As a result, with the help of the adjusted tolerance, the FE for each reconstructed frequency band is calculated according to (5) and can be expressed as (16) and (17):

(16) $$FE_{A_{L}}=\mathrm{FE}\left({\tilde{x}}_{A_{L}},\;m,{\;r}_{A_{L}},\;n\right)$$

(17) $$FE_{D_{i}}=\mathrm{FE}\left({\tilde{x}}_{D_{i}},\;m,{\;r}_{D_{i}},\;n\right),\;i=1,\;2,\;\ldots ,\;L$$

Lastly, the FE values from all frequency bands are combined to form the HMFE, aiding in the differentiation of lie state from frequency characteristics (18):

(18) $$\mathrm{HMFE}=\left[FE_{A_{L}},\;FE_{D_{L}},\;FE_{D_{L-1}},\;\ldots ,\;FE_{D_{1}}\right]$$

### 2.2. Classification Method

To establish a lightweight entropy-based method, different classifiers (SVM, kNN, NB, and LDA) are employed to train and test the extracted multi-scale entropy features, which are then fused into a vector form to serve as the input data for the classifiers. After thoroughly assessing the classifiers for discriminating between lie and truth states, the most suitable one for lie detection can be determined in the proposed method.

First, SVM involves identifying a hyperplane that maximizes the classification margin, which separates data points belonging to different classes [31]. By maximizing this margin, SVM can achieve improved generalization ability, performing well even with limited training samples. In this study, two kernel functions are evaluated: linear (SVM-Linear) and RBF (SVM-RBF), which aim to investigate the influence of different decision boundary complexities on lie detection. The linear kernel is proper for linearly separable data, allowing for the construction of simple decision boundaries. On the other hand, the RBF kernel maps the original data to a higher-dimensional space, demonstrating good adaptability in complex data classification.

Second, kNN is an instance-based method, categorized as a non-parametric classification in machine learning [32]. In k-NN, for a sample with an unknown class, it identifies the k-nearest neighbors from the training set. The class of the unknown sample is then determined by weighted averaging based on the labels of these neighbors. In this study, two k values (k = 3 and k = 5) are assessed. The choice of these small, odd integers is a standard practice in binary classification to avoid tied votes. This selection also allows for a direct exploration of the bias-variance trade-off in a computationally efficient manner, as k = 3 results in a model with lower bias and higher variance, making it highly adaptive to local data structures, while k = 5 provides a smoother decision boundary with lower variance, which can enhance generalization. Given the goal of evaluating a lightweight system and the nature of the dataset, these values serve as effective and representative benchmarks for the kNN’s performance.

Third, LDA is a classical linear classification that finds one or more linear combinations of features such that the projections of data from different classes onto these linear combinations achieve maximum between-class variance and minimum within-class variance [33]. It assumes identical covariance matrices for all classes, offers high computational efficiency, and provides easily interpretable results, which makes it particularly well-suited for modeling and predicting classes given feature values.

Next, NB classifier assumes that all features are conditionally independent given the class [34]. Although this naive assumption rarely holds perfectly in real-world scenarios, it is favored for its simplicity, efficiency, adaptability to small sample sizes, and exemplary performance in EEG-based applications.

Moreover, accuracy is used to evaluate these classifiers. It denotes the proportion of correctly classified samples relative to the total number of samples (19):

(19) $$\mathrm{Accuracy}=\frac{\mathrm{TP}+\mathrm{TN}}{\mathrm{TP}+\mathrm{TN}+\mathrm{FP}+\mathrm{FN}}$$

where true positive (TP) means the number of samples whose true label is lie and are correctly predicted as lie, true negative (TN) represents the number of samples whose true label is truth and are correctly predicted as truth, false positive (FP) indicates the number of samples whose true label is truth but are incorrectly predicted as lie, and false negative (FN) means the number of samples whose true label is lie but were incorrectly predicted as truth.

Please note that while other metrics such as sensitivity, specificity, and the complete confusion matrix are often crucial for the method evaluation, accuracy is selected as the primary metric in this study due to the balanced nature of the experimental dataset, where each subject provides an equal number of trials for the ‘lie’ and ‘truth’ states (25 trials per class). Thus, in a perfectly balanced binary classification task, accuracy is a direct and unbiased metric to show overall performance, as a majority class does not skew it and can provide a straightforward measure of how well the classifiers can distinguish between the two different states accordingly. More details about the experimental dataset are described subsequently.

### 2.3. Experimental Dataset

In this study, method validation was conducted using the publicly available LieWaves dataset [35], which is specifically designed to offer high-quality EEG signals for neurophysiology-based lie detection by analyzing individual brain activities during lie and truth states. The data acquisition for the LieWaves dataset involves recruiting 27 healthy subjects, all are students or faculty members from the Faculty of Technology at Karadeniz Technical University, Türkiye. Their average age is 23.1 years, and none has any known health issues. Before the experiments, all subjects were fully informed about the content and procedures and voluntarily signed an ethics-informed consent form.

**Figure 2:**
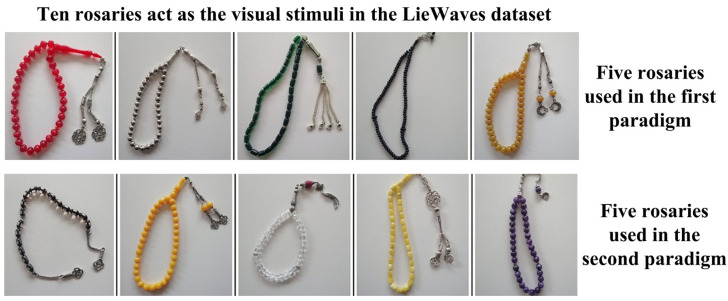
Ten rosaries act as the visual stimuli in the LieWaves dataset.

During the experiment, each subject underwent two independent paradigms designed to elicit truth and lie states using a concealed information test-style protocol. To prevent fatigue effects, ten different rosaries were used as visual stimuli, with five allocated to each paradigm, as shown in Figure 2.

Regarding the truth paradigm, subjects were first familiarized with a set of five rosaries. During the EEG recording session, their tasks were to acknowledge recognition when these items were presented truthfully. Concerning the lie paradigm, subjects were shown a different set of five rosaries and were instructed to select one as a critical item. So, their tasks were to deceptively deny having seen this specific item when it was presented, constituting the lie condition accordingly.

For both paradigms, each subject completed 25 trials (five rosaries, each repeated five times). Each trial lasted three seconds, consisting of a one-second black screen prompt followed by a two-second visual stimulus presentation. Moreover, all subjects provided their responses, either a truthful expression or a deceptive one, by pressing a designated key on a keyboard. Consequently, 75 s (25 trials × 3 s) of EEG signals were collected from each subject for each paradigm.

The data acquisition was performed using an Emotiv Insight headset, which includes the five channels (AF3, T7, PZ, T8, and AF4). Particularly, its low number of channels and wireless, portable design provided convenience during EEG data collection. Additionally, it exhibited an internal sampling rate of 2048 Hz. However, the data has been downsampled internally before output, so the dataset provided data at a final sampling rate of 128 Hz. Meanwhile, to ensure data quality, raw EEG signals were pre-processed, which involved removing the direct current (DC) offset and then applying a band-pass filter with a frequency range of 0.5–45 Hz. This filtering step serves to eliminate low-frequency drifts (below 0.5 Hz) and high-frequency noise, including 50/60 Hz power-line interference, while preserving the primary EEG frequency bands of interest (delta, theta, alpha, and beta) for analysis. While filtering helps attenuate several high-frequency muscle activities, dedicated artifact removal techniques are necessary to address muscle artifacts. In short, the dataset has dimensions of 27 × 2 × 5 × 75, where ‘27’ represents the number of subjects, ‘2’ denotes the number of experimental paradigms (lie and truth), ‘5’ means the number of channels, and ‘75’ indicates the duration of each experiment in seconds. Based on this, two types of experiments (the subject-dependent experiment and the cross-subject experiment) were conducted, with each employing the feature vector extracted from the same EEG channel as input data. The details are as follows:

#### 2.3.1. Subject-Dependent Experiment

The subject-dependent experiment primarily evaluates the proposed method for distinguishing between lie and truth states within the same individual and for identifying the representative channel of each subject. To this end, the 50 trials per subject are assessed by leave-one-out cross-validation (LOOCV). In this procedure, one trial serves as the test sample, and the remaining 49 trials are used for training. This process is iterated 50 times, such that each trial is used as the test sample exactly once. The final accuracy is the average performance across all 50 iterations. Additionally, 10-fold cross-validation is also performed. Through the two cross-validation methods, the accuracy of each channel using different classifiers can be fully evaluated for each subject.

#### 2.3.2. Cross-Subject Experiment

The cross-subject experiment aims to assess the generalization capability of the proposed method on unseen subject data, which is vital when dealing with new users in practical cases. To this end, the leave-one-subject-out cross-validation (LOSOCV) is adopted. Specifically, in each iteration, samples from one subject (including all their trials) are selected as the test set, while all trials from the remaining 26 subjects constitute the training set. This process is iterated until all 27 subjects have served as the test set once. Finally, the average accuracy across all 27 iterations is calculated to reflect the average performance. Simultaneously, each channel and its corresponding accuracy are analyzed for each subject when serving as the test set, which also facilitates the channel selection.

## 3. Experimental Results

The experimental results are analyzed for two scenarios: subject-dependent and cross-subject. A thorough analysis of different channels is also conducted. Please note that for convenient presentation of the results, ‘C1’ to ‘C6’ sequentially refer to the six types of classifiers evaluated, where C1 is for SVM-Linear, C2 is for SVM-RBF, C3 is for kNN-3, C4 is for kNN-5, C5 is for NB, and C6 is for LDA. Meanwhile, ‘S1’ to ‘S27’ denote the 27 subjects in the dataset.

### 3.1. Subject-Dependent Results

Table 1 shows the accuracies for different classifiers across subjects under LOOCV and 10-fold cross-validation, where the best results are underlined in bold, and ‘SD’ refers to standard deviation.

From Table 1, it can be observed that in the subject-dependent experiment, C6 (LDA) achieves the highest average accuracies under two cross-validation methods, at 82.74% and 82.00%, respectively, which indicates that the proposed multi-scale entropy approach combined with LDA exhibits satisfactory performance in distinguishing lie and truth states within individuals. It is noteworthy that considerable variations in detection accuracy exist in several subjects. For instance, S2, S9, S13, and S25 consistently attained accuracies of up to 98% across both cross-validation methods, demonstrating high discriminability of their individual EEG signals in lie detection. However, S17, S19, and S23 show lower accuracies. Such disparity reflects inter-individual heterogeneity in physiological responses. To further investigate the specific performance of each subject under the LDA, particularly to select the representative channels, Table 2 shows the channels that yield the highest accuracy by utilizing the LDA classifier for all 27 subjects.

In Table 2, the statistical results indicate that PZ and T7 are representative, as their occurrences are the most frequent among all channels. From a neuroscience perspective, the PZ is commonly associated with cognitive control, while the T7 is related to language processing, memory retrieval, and auditory processing [36,37]. The involvement of PZ reflects the increased demand for executive functions, such as response inhibition (suppressing the truth) and cognitive monitoring (managing the narrative of the lie). Concurrently, the involvement of T7 highlights its crucial role in accessing relevant semantic knowledge and potentially rehearsing the false statement internally. Together, their prominences reveal that lying is not a localized process but instead involves a coordinated network that needs both executive control over behavior and complex semantic operations, offering neurophysiological support for lie detection.

### 3.2. Cross-Subject Results

Table 3 lists the accuracies of the cross-subject experiment using different classifiers under LOSOCV, where the best results are underlined in bold. ‘SD’ refers to standard deviation.

Table 3 reveals that in the cross-subject experiment, C2 (SVM-RBF) achieves the highest average accuracy of 64.07%. Compared to the results in the subject-dependent experiment (approximately 82%), the cross-subject accuracies are generally lower, which aligns with expectations, as generalizing across individual data is more challenging, requiring the classifier to learn features unaffected by inter-individual variability. Hence, there is a trade-off between the generalization and accuracy. Now, to further analyze the channel selection in the cross-subject experiment, Table 4 lists the channels that yield the highest accuracy when using the SVM-RBF classifier.

From Table 4, the statistical results indicate that PZ and T7 channels also exhibit a prominent occurrence as representatives, which support the potential importance of PZ and T7 channels in lie detection, as these two channels consistently provide relatively stable discriminative information through entropy-based analyses, even under the cross-subject generalization assessment, which corroborates the roles these two specific channel locations play in processing cognitive state related to lying.

### 3.3. Channel Selection Results

**Figure 3:**
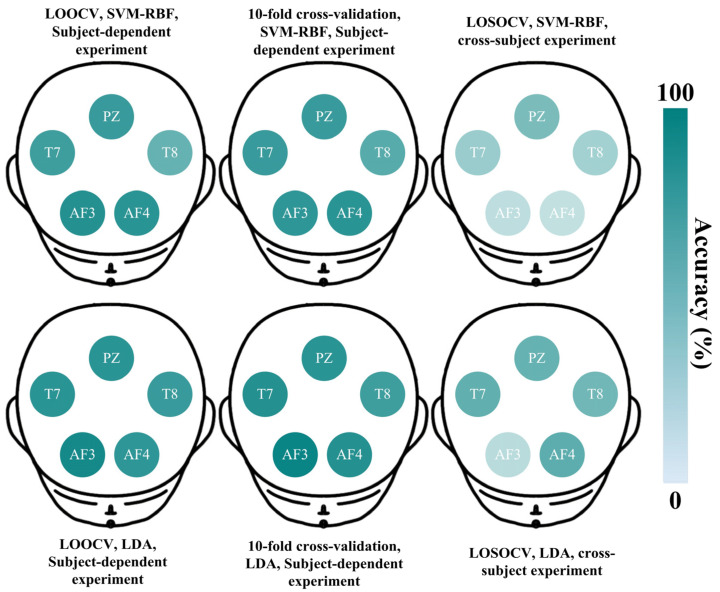
Lie detection accuracies across five channels for the two classifiers (SVM-RBF and LDA) under three cross-validation methods (LOOCV, 10-fold cross-validation, and LOSOCV). The data source is from S4 in the LieWaves dataset.

To thoroughly analyze channel selection in EEG-based lie detection, the accuracies across channels for the two best classifiers (SVM-RBF and LDA) are investigated in detail under three cross-validation methods (LOOCV, 10-fold cross-validation, and LOSOCV). As an example, Figure 3 illustrates the scalp maps from subject-dependent and cross-subject results of S4.

**Figure 4:**
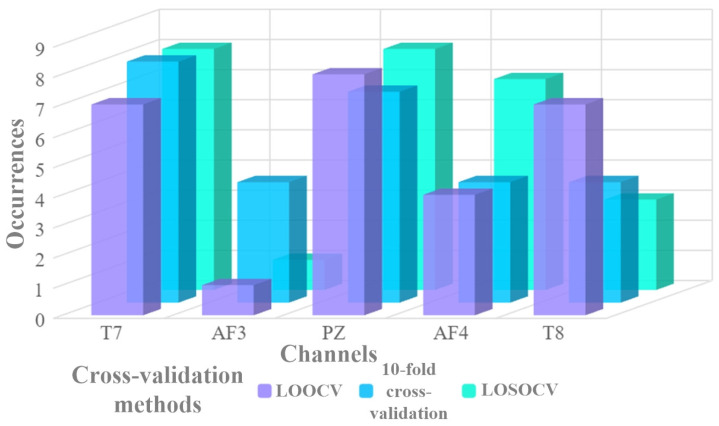
Statistical results of the representative channels under subject-dependent and cross-subject experiments for EEG-based lie detection, where PZ and T7 channels show higher cumulative occurrences in this context.

In Figure 3, the deeper colors indicate higher accuracy. In the subject-dependent experiment of S4, both SVM-RBF and LDA exhibit satisfactory accuracies across five channels. The PZ and T7 channels consistently demonstrate better discriminative abilities in lie detection, which aligns with the results in Table 2. In the cross-subject experiment, the overall results indicate a general decrease in accuracy. While the PZ and T7 channels still show relatively deeper colors, further demonstrating their importance for lie detection. Based on this, the representative channels from both subject-dependent and cross-subject experiments are statistically illustrated in Figure 4.

Figure 4 presents the cumulative occurrences of each EEG channel serving as the representative channel across all cross-validation methods. It is evident that the T7 and PZ channels appear most frequently as the best-performing channels. It is also noteworthy that the T8 channel demonstrates a strong performance, although its cumulative occurrence is lower than that of T7 and PZ. The prominence of such channels, particularly T7 and PZ, not only highlights key brain regions of focus for lie detection but also provides empirical evidence for channel selection. In developing portable EEG-based lie detection devices with the fewest possible channels, the higher incidence rate of T7 and PZ makes them the primary candidates.

To further investigate the potential of a multi-channel configuration and to provide a direct comparison against the single-channel findings, an additional analysis is conducted using two-channel combinations. Here, the three channels that performed most prominently in the single-channel analysis (PZ, T7, and T8) are chosen to create feature sets for all their pairwise combinations (PZ-T7, PZ-T8, and T7-T8). The classification is then repeated using the same validation schemes and best-performing classifiers. The detailed results are presented in Table 5. ‘SD’ refers to standard deviation.

## 4. Discussion

To contextualize the findings and evaluate the practical advantages of this study, several recent works in EEG-based lie detection are considered, as summarized in Table 6. It is crucial to emphasize that a direct comparison of accuracy between these studies is inappropriate, as they employ different datasets, subjects, and specific experimental protocols. Therefore, the purpose of Table 6 is not to claim numerical superiority but to highlight the key methodological advantage, which serves as a methodological summary to frame this study’s contribution in terms of efficiency and hardware requirements, contrasting the single-channel approach with the multi-channel systems used in other conceptually similar works.

In this study, the discrimination between lie and truth can be achieved using a single-channel data based on the multi-scale entropy approach combined with conventional classifiers. Within the subject-dependent experiment, satisfactory performance is demonstrated, with the LDA classifier yielding average accuracies of 82.74% (LOOCV) and 82.00% (10-fold cross-validation). These results reveal that the multi-scale entropy features effectively capture the subtle neurophysiological behavior associated with the lie state. The prominence of the PZ (midline parietal) and T7 (left temporal) channels across experiments strongly suggests their critical involvement in the neurocognitive substrates of lie detection. However, it is essential to acknowledge the strong performance of the T8 channel as well, which suggests that the right temporal lobe plays a complementary role, indicating that a bilateral temporal network is involved in deception. The decision to prioritize PZ and T7 is driven by the practical goal of designing a system with the minimum number of channels necessary for robust performance. For a hypothetical two-channel device, PZ and T7 are the empirically supported optimal choices from our findings. Nonetheless, the notable results from T8 open an engaging way for future research into whether a three-channel configuration (PZ, T7, and T8) would yield a significant improvement in accuracy. As mentioned, the PZ channel is implicated in attention, working memory, and context integration, while the T7 channel is associated with semantic processing, both of which are key processes in deception. Such findings imply the proposed entropy-based approach contributes to representative channel selection, providing insightful understandings in the field of electrophysiological signals.

Moreover, when benchmarked against established EEG-based lie detection techniques, the significance of the single-channel results found in this study becomes evident. Although better accuracies have been reported using multi-channel approaches, they require greater computational resources, complex spatial filtering, and a larger number of electrodes placed on the scalp for recordings. In contrast, the proposed method achieves around 82% accuracy using only single-channel data, demonstrating a trade-off. An additional investigation reinforces this consideration, where combining features from the best-performing channels (e.g., PZ and T7) surprisingly led to a decrease in accuracy, dropping from 82.74% to 77.56% in the subject-dependent LOOCV test. This counterintuitive result is likely due to the curse of dimensionality, since the increased feature space hinders the classifier’s performance given the limited number of trials, which also provides compelling evidence that a single, well-selected channel is not merely a compromise for efficiency but is, in fact, the optimal approach for maximizing accuracy with the proposed multi-scale entropy features. Consequently, the representative channel selection performed in this study represents an advancement in computational cost and hardware deployment, reducing redundant data acquisition while maintaining satisfactory performance for individual-centric daily applications.

Next, an interesting observation from the subject-dependent results (Table 1) is that the kNN-3 classifier occasionally outperforms the kNN-5 classifier for several subjects. While one might typically expect performance to improve with a slightly larger k, this phenomenon can be explained by the bias-variance trade-off. A smaller k value, such as 3, creates a highly flexible decision boundary that is sensitive to the local structure of the data (i.e., low bias, high variance). For subjects whose EEG feature distributions for lie and truth are complex and intricately separated, this adaptive boundary may be superior at capturing the fine distinctions. In contrast, k = 5 enforces a smoother decision boundary, which, while potentially more robust against noise, might oversmooth these critical separating patterns for certain individuals, leading to a marginal decrease in accuracy. Thus, such variations reveal the inter-subject variability in neurophysiological responses and the challenge of finding a one-size-fits-all model.

Finally, the generalization needs to be enhanced within the cross-subject experiment, where an average accuracy of 64.07% is attained using the SVM-RBF classifier. While this result highlights the inherent difficulty of subject-independent lie detection and the influence of individual neurophysiological variability, it concurrently validates the transferable discriminative ability of the extracted entropy-based features. The selection of PZ and T7 as representative channels also provides valuable insights into the field of neuroscience. It can be said that the convergence of single-channel EEG processing with electrophysiological discoveries establishes a suitable pathway toward developing portable devices, further lowering deployment barriers while advancing the fundamental understanding of the neural dynamics underlying lie states.

## 5. Conclusions

This study establishes an EEG-based lie detection method by extracting multi-scale entropy with representative channel selection. Designed to comprehensively quantify EEG signal complexity across both temporal and spectral domains, the proposed entropy-based approach overcomes limitations inherent in traditional single-scale and coarse-graining methods. The experimental evaluation on the LieWaves dataset demonstrates satisfactory performance in the subject-dependent experiment, with the LDA classifier achieving an accuracy of approximately 82%. Then, the cross-subject experiment proves more challenging, yielding an accuracy of 64.07% using the SVM-RBF classifier. Moreover, the representative channels are found as PZ and T7, which are involved in insightful neurocognitive processes of lie detection, providing empirical evidence for optimizing channel selection on the scalp for practical applications. Future work will focus on enhancing the robustness of the multi-scale entropy approach in terms of cross-subject generalization capabilities, particularly in real-world scenarios with environmental noise and increased cognitive load, advancing the evolution of EEG-based applications.

## Figures and Tables

**Table 1:** Lie detection accuracy (%) using different classifiers in the subject-dependent experiment under LOOCV and 10-fold cross-validation.

Subject	LOOCV Accuracy (%)						10-Fold Cross-Validation Accuracy (%)					
	C1	C2	C3	C4	C5	C6	C1	C2	C3	C4	C5	C6
S1	60	72	80	74	72	84	62	74	80	72	72	86
S2	98	98	94	92	98	98	98	98	96	92	98	98
S3	70	72	66	72	76	68	58	74	64	72	74	68
S4	88	86	86	88	84	92	88	84	86	86	84	96
S5	74	76	74	70	72	70	74	76	70	70	74	68
S6	68	68	54	62	68	74	66	68	60	60	66	64
S7	86	88	84	88	86	84	88	88	82	88	86	82
S8	72	74	66	74	76	68	74	76	66	78	76	70
S9	98	98	98	98	98	98	98	98	98	98	98	98
S10	80	80	78	80	82	82	82	80	80	82	80	78
S11	66	78	72	68	72	82	68	78	68	72	72	76
S12	96	96	96	96	96	94	96	94	96	96	96	92
S13	98	98	98	98	98	98	98	98	98	98	98	98
S14	80	86	78	82	80	80	84	82	78	80	80	80
S15	80	78	74	82	82	88	80	78	80	82	82	88
S16	62	74	70	76	64	76	64	70	72	80	66	82
S17	58	44	58	60	60	82	60	54	56	58	58	80
S18	92	94	90	94	94	88	94	92	90	94	92	86
S19	60	52	56	58	62	66	60	58	66	60	62	68
S20	78	78	72	72	80	72	78	78	72	74	80	66
S21	82	82	84	82	84	86	82	80	84	82	84	82
S22	86	86	88	86	88	90	86	86	90	86	88	90
S23	60	62	64	66	60	62	56	58	66	66	56	66
S24	70	68	66	70	74	72	66	70	64	68	78	74
S25	98	94	94	98	98	98	98	94	94	96	98	98
S26	94	94	96	96	96	90	92	94	94	94	96	90
S27	86	88	88	82	90	92	84	92	86	86	88	90
Mean	79.26	80.15	78.67	80.15	81.11	**82.74**	79.04	80.44	79.11	80.37	80.81	**82.00**
SD	13.45	13.83	13.43	12.35	12.40	10.87	14.04	12.51	12.82	12.14	12.60	11.22

**Table 2:** The channels that yield the highest accuracy when utilizing the LDA classifier for all 27 subjects in the subject-dependent experiment.

Subject	LOOCV		10-Fold Cross-Validation	
	Accuracy (%)	Channel	Accuracy (%)	Channel
S1	84	T7	86	T7
S2	98	T7	98	T7
S3	68	T8	68	T8
S4	92	T7	96	T7
S5	70	T7	68	AF3
S6	74	T8	64	PZ
S7	84	AF3	82	T7
S8	68	PZ	70	PZ
S9	98	T7	98	T7
S10	82	AF4	78	AF4
S11	82	AF4	76	AF4
S12	94	PZ	92	PZ
S13	98	T8	98	T8
S14	80	T7	80	T7
S15	88	AF4	88	AF4
S16	76	AF4	82	AF4
S17	82	PZ	80	PZ
S18	88	T8	86	AF3
S19	66	T8	68	AF3
S20	72	PZ	66	PZ
S21	86	T8	82	T8
S22	90	PZ	90	T7
S23	62	PZ	66	PZ
S24	72	T7	74	T7
S25	98	PZ	98	PZ
S26	90	T8	90	T8
S27	92	PZ	90	AF3

**Table 3:** Lie detection accuracy (%) using different classifiers in the cross-subject experiment under LOSOCV.

Subject	LOSOCV Accuracy (%)					
	C1	C2	C3	C4	C5	C6
S1	74	64	66	60	62	64
S2	64	60	62	64	54	66
S3	54	66	58	62	54	58
S4	66	52	54	46	84	64
S5	56	66	68	70	54	58
S6	56	68	60	56	66	58
S7	68	76	58	66	54	62
S8	52	62	58	54	56	62
S9	28	34	52	52	42	36
S10	68	76	52	58	54	66
S11	50	60	58	62	56	54
S12	66	80	62	66	94	62
S13	80	68	60	68	54	72
S14	58	78	62	62	58	58
S15	58	54	50	58	52	58
S16	60	72	60	48	56	70
S17	64	62	56	54	54	62
S18	72	80	70	70	72	72
S19	52	58	64	72	46	54
S20	74	76	64	78	78	74
S21	68	64	58	58	68	58
S22	60	62	66	68	74	68
S23	54	64	66	66	60	60
S24	60	50	64	64	54	60
S25	58	68	60	70	64	62
S26	56	58	62	60	62	58
S27	54	52	56	56	52	54
Mean	60.37	**64.07**	60.22	61.78	60.52	61.11
SD	10.09	10.49	5.00	7.55	11.60	7.47

**Table 4:** The channels that yield the highest accuracy when using the SVM-RBF classifier in the cross-subject experiment.

Subject	Accuracy (%)	Channel
S1	64	AF3
S2	60	PZ
S3	66	T7
S4	52	PZ
S5	66	AF4
S6	68	T8
S7	76	T8
S8	62	PZ
S9	34	T7
S10	76	AF4
S11	60	T7
S12	80	PZ
S13	68	AF4
S14	78	T7
S15	54	AF4
S16	72	AF4
S17	62	T7
S18	80	PZ
S19	58	AF4
S20	76	PZ
S21	64	T8
S22	62	T7
S23	64	PZ
S24	50	PZ
S25	68	T7
S26	58	AF4
S27	52	T7

**Table 5:** Lie detection accuracies (%) of the multi-channel configuration using SVM-RBF and LDA with different cross-validation methods.

Subject	10-Fold Cross-Validation						LOSOCV					
	SVM-RBF			LDA			SVM-RBF			LDA		
	T7-PZ	T7-T8	PZ-T8	T7-PZ	T7-T8	PZ-T8	T7-PZ	T7-T8	PZ-T8	T7-PZ	T7-T8	PZ-T8
S1	76	72	70	68	56	60	50	58	48	48	54	54
S2	94	98	96	96	98	98	54	52	62	50	54	52
S3	58	56	58	74	72	62	50	34	50	52	46	50
S4	80	70	84	84	78	78	40	36	42	62	62	54
S5	62	74	70	64	64	68	46	72	56	58	50	52
S6	54	42	58	54	52	60	52	44	50	60	58	54
S7	80	78	74	74	66	64	50	42	52	64	56	76
S8	82	70	54	74	54	54	38	50	52	48	62	60
S9	98	86	98	98	92	98	20	26	60	20	18	20
S10	82	68	86	68	74	82	60	72	54	62	66	72
S11	64	78	76	56	72	70	58	54	54	56	58	48
S12	96	52	96	84	64	86	48	62	46	68	64	60
S13	94	98	96	98	98	98	56	62	50	74	38	62
S14	62	70	68	68	56	72	42	32	50	46	48	32
S15	68	58	72	78	86	90	46	46	50	50	54	52
S16	66	80	72	68	74	68	54	52	56	56	60	64
S17	60	40	42	68	56	68	56	60	64	60	62	56
S18	84	70	84	82	74	82	46	34	36	52	60	72
S19	52	58	40	52	66	54	54	50	48	50	56	50
S20	78	56	78	72	60	76	56	40	52	56	36	60
S21	76	82	86	86	78	84	34	50	50	62	56	62
S22	74	72	72	86	74	84	62	42	52	56	48	46
S23	54	68	70	76	64	70	58	58	58	52	42	52
S24	70	74	70	76	60	68	54	42	46	60	42	56
S25	94	94	94	98	98	98	50	44	50	72	42	56
S26	82	70	86	80	60	68	20	34	32	24	42	18
S27	94	84	82	94	84	94	50	54	52	54	48	50
Mean	75.33	71.04	75.26	76.89	71.48	76.07	48.29	48.22	50.81	54.51	51.18	53.33
SD	14.20	14.77	15.57	13.14	13.92	13.85	10.53	11.90	6.89	11.76	10.70	13.25

**Table 6:** A methodological summary of recent EEG-based lie detection works.

Work	Dataset	Channels	Subjects	Main Methodologies		Accuracy (%)
				Feature	Classifier	
Edla et al. [38]	Private	16	10	CSP-16	SNN	79.69
Wang et al. [39]	Private	8	10	RCIT		87.13
Rahimi Saryazdi et al. [40]	Private	33	22	WDPVG	SVM	86.25
					kNN-5	66.64
					DT	82.46
Bablani et al. [41]	Private	16	9	Hybrid	Ensemble	84.70
Kang et al. [42]	Private	64	36	PMI	FCN	88.50
This study	LieWaves	1	27	Multi-scale entropy	LDA	82.74

CSP-16: common spatial pattern with order 16; SNN: spiking neural network; RCIT: rapid serial visual presentation-based concealed information test framework; WDPVG: weighted dual perspective visibility graph; DT: decision tree; Hybrid: various features such as amplitude, complexity, mobility frequency, power, and wavelet. Ensemble: voting by LDA, SVM, MLFNN (multilayer feedforward neural network); PMI: partial mutual information; FCN: functional connectivity network classifier.

## Data Availability

The datasets generated and/or analyzed during the current study are available at https://github.com/zyzc75/ME-Lie-Detection (accessed on 1 September 2025).
